# A rare case of bilateral bronchial foreign body

**DOI:** 10.12669/pjms.312.6060

**Published:** 2015

**Authors:** ZhiGang Zhao, Qian Gao, PengLong Song

**Affiliations:** 1 ZhiGang Zhao, MD, Department of Otorhinolaryngology/Head and Neck Surgery, The First Affiliated Hospital, Harbin Medical University, Harbin 150010, Heilongjiang Province, People’s Republic of China; 2 Qian Gao, MD, Department of Otorhinolaryngology/Head and Neck Surgery, The First Affiliated Hospital, Harbin Medical University, Harbin 150010, Heilongjiang Province, People’s Republic of China; 3 PengLong Song, MD, Department of Otorhinolaryngology/Head and Neck Surgery, The First Affiliated Hospital, Harbin Medical University, Harbin 150010, Heilongjiang Province, People’s Republic of China

**Keywords:** Foreign bodies, Bronchi

## Abstract

We present the case of a 7-year-old male patient with bilateral bronchial cocklebur fruit aspiration, which he sustained while playing. The patient presented with a triad of cough, wheezing, and decreased breath sounds (decreased in the right lung and absent in the left). These symptoms led to a diagnosis of bilateral bronchial foreign body, which was confirmed by computed tomography three-dimensional reconstruction of the bronchial tree. The patient was on the verge of death during operation but was ultimately rescued. Our therapeutic experience in treating this case of bilateral bronchial cocklebur fruit aspiration may provide a good reference for others.

## INTRODUCTION

Foreign body aspiration into the tracheobronchial tree is known to occur in all age groups,^1^,^2^ but children aged 1–3 years are the most susceptible.^1^ The current mortality rate from foreign body aspiration is between 0% and 1.8% according to various studies.^3^,^4^ Early diagnosis and prompt retrieval of a tracheobronchial foreign body are important in reducing the incidence of complications and mortality. Although bronchoscopy under general anesthesia augmented by topical anesthesia is a very safe and effective procedure for a patient with a tracheobronchial foreign body, it is quite risky for patients requiring bilateral bronchial foreign body removal.^1^ Various studies estimate the current morbidity rate from bilateral bronchial foreign body as between 0.24% and 2%.^1^ Although much has been written in the literature about bronchial foreign body, few reports of physicians’ therapeutic experience about bilateral bronchial foreign body are available for guidance because of the low morbidity rate. We therefore present our experience of a case of bilateral bronchial foreign body.

## CASE REPORT

**Fig.1:**
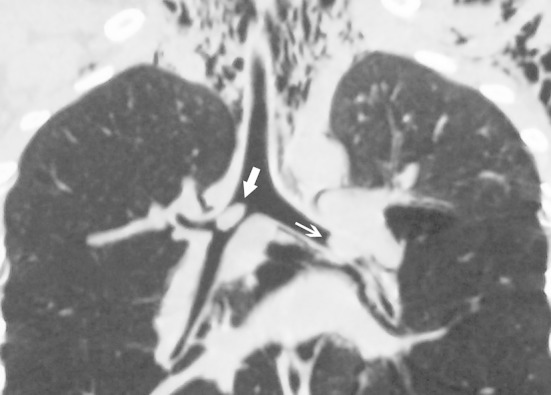
Three-dimensional reconstruction chest CT confirmed bilateral bronchial foreign body. The white arrow indicates the foreign body in the right bronchus and the black arrow indicates the foreign body in the left bronchus.

A 7-year-old male patient with a 48-hour history of cocklebur fruit aspiration was admitted to our hospital diagnosed with bilateral bronchial foreign body. The cocklebur fruit (genus *Xanthium*) is a seed pod measuring approximately 1–2 cm that is covered with stiff, hooked spines. The patient presented with the typical triad of cough, wheezing, and decreased breath sounds (decreased in the right lung and absent in the left). Examination revealed orthopnea and lip and skin cyanosis. Three-dimensional reconstruction of the bronchial tree confirmed the diagnosis of bilateral bronchial foreign body (Fig.1). Retrieval of the bronchial foreign bodies was performed shortly after admission under combined intravenous anesthesia and high-frequency positive-pressure ventilation. We first attempted to retrieve the cocklebur fruit in the right main bronchus because of its location near to bifurcation of the trachea and relative accessibility. However, during the operation, the cocklebur fruit in the right bronchus was pushed to a deeper level by the trielcon. The rigid bronchoscope was then passed into the right bronchus followed the foreign body pushed even further, and the patient’s blood oxygen saturation declined rapidly from 90% to 15%. At that time, the only option that would save the child’s life was to retrieve the right bronchial foreign body as quickly as possible. Fortunately, we were able to extract the foreign body in the next 30 seconds using a crocodile clamp, and oxygen saturation quickly returned to 90%. We then successfully extracted the left bronchial cocklebur fruit and the child was discharged on postoperative day two.

## DISCUSSION

The literature describes rigid bronchoscopy as the main technique used in the removal of bronchial foreign bodies and thus as an important tool for increasing ventilation safety and for manipulation in children’s airways.^5^,^6^ In our opinion, rigid bronchoscopy is mandatory in the removal of a bronchial foreign body. However, other useful techniques, such as flexible bronchoscopy, suspension laryngoscopy and fluoroscopy, deserve attention as part of medical training.^5^

Three-dimensional reconstruction chest computed tomography (CT) is an accurate and non-invasive examination used in the diagnosis of tracheobronchial foreign body. It is generally known that three-dimensional chest CT was more sensitive than chest x-ray in detecting the presence of aspirated foreign bodies in children.^7^,^8^ The superior sensitivity and short time required for CT can reduce delays in diagnosis. In the present case, three-dimensional reconstruction chest CT not only rapidly confirmed the presence of foreign body, but also directly and accurately displayed the position, size, shape, and degree of obstruction, which are crucial to the successful operation.

Treatment of airway foreign body in pediatrics is challenging because it requires skilful rigid bronchoscopy with anesthesia under the condition of a compromised ventilation-perfusion ratio. When bronchoscopy for bilateral foreign body fails, thoracotomy may need to be performed, and for bilateral complete obstruction, emergency tracheostomy is done.^9^ The operator’s skill and experience in dealing with such cases are very important.^10^ In this case, the patient almost died during the operation, but the treatment outcome was ultimately satisfactory. We analyzed the case retrospectively and conjectured that retrieving the left lung cocklebur fruit first may have been safer and more effective than attempting to retrieve the one on the right. We judged the effect of the foreign body to be greater on the left lung, where there were absent breath sounds, than on the right, where there were decreased breath sounds. In such a case, the patient may be depending mainly on the remnant pulmonary function. During our patient’s operation, we attempted to retrieve the right cocklebur fruit in one pass but were unsuccessful. The cocklebur fruit was pushed to a deeper level by the trielcon, and the rigid bronchoscopy that followed obstructed the right lung entirely, from which the patient almost died. We conclude that when faced with bilateral bronchial foreign body patients, unless the foreign body causing less obstruction can be removed in one stroke, the foreign body most completely obstructing the lung should be removed first.

**Fig.2:**
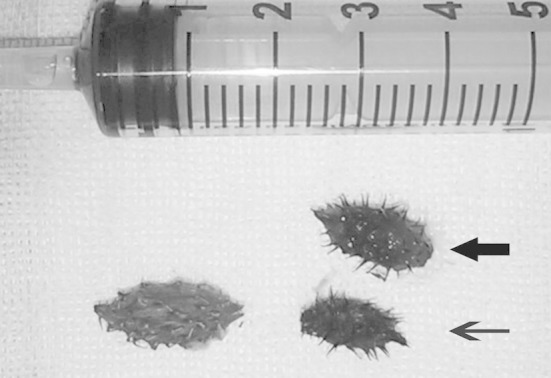
The bitten cocklebur fruit removed from bilateral bronchi. The white arrow indicates the bitten cocklebur fruit from the left bronchus and the black arrow indicates the bitten cocklebur fruit from the right bronchus.

In fact, to remove the foreign body in one stroke, we had simulated the procedure in vitro preoperatively and concluded that the entire cocklebur fruit could be grasped easily and tightly by the trielcon. We believed that the right cocklebur fruit, which was near the bifurcation of the trachea, could be extracted in one pass. However, during the operation, the right cocklebur fruit was removed not by the trielcon but by a crocodile clamp; switching instruments wasted about half a minute and the patient almost died. To our surprise, the foreign body in the right lung was not a whole cocklebur fruit but a large portion of it (Fig.2). Because it was difficult to believe that the pediatric patient would put the cocklebur fruit, with its stiff spines, in his mouth voluntarily, a detailed case history was taken after the operation. In fact, one of the patient’s playmates had forcefully stuffed a cocklebur into his mouth during a quarrel. The patient inadvertently bit in two and aspirated both parts. The case reminds us of the importance of acquiring a detailed case history preoperatively.

## CONCLUSION

We believe that when clinical presentation strongly suggests a tracheobronchial foreign body, especially bilateral bronchial foreign body, three-dimensional reconstruction chest CT is indispensable for a successful operation. Furthermore, when faced with bilateral bronchial foreign body patients, unless the foreign body causing less obstruction and can be removed in one stroke, the foreign body most completely obstructing the lung should be removed first.
